# Soil organic carbon, total nitrogen stocks and CO_2_ emissions in top- and subsoils with contrasting management regimes in semi-arid environments

**DOI:** 10.1038/s41598-023-28276-x

**Published:** 2023-01-20

**Authors:** Chukwuebuka C. Okolo, Girmay Gebresamuel, Amanuel Zenebe, Mitiku Haile, Jephter E. Orji, Chinyere B. Okebalama, Chinedu E. Eze, Emmanuel Eze, Peter N. Eze

**Affiliations:** 1 Department of Land Resources Management and Environmental Protection, Mekelle University, P. O. Box 231, Mekelle, Ethiopia; 2 Institute of Climate and Society, Mekelle University, P. O. Box 231, Mekelle, Ethiopia; 3 Department of Natural Resources Management, Jimma University, P. O. Box 378, Jimma, Ethiopia; 4 Department of Geoscience, Geo-Biosphere Interaction Group, Eberhard Karls University Tübingen, 72076 Tübingen, Germany; 5 Department of Earth and Environmental Science, Botswana International University of Science and Technology, Private Bag 16, Palapye, Botswana; 6 Department of Agriculture, Alex Ekwueme Federal University Ndufu-Alike, Ikwo, Nigeria; 7 Department of Soil Science, Faculty of Agriculture, University of Nigeria Nsukka, Nsukka, Nigeria; 8 Ruhr University Bochum, Bochum, Germany; 9 Department of Agronomy, Michael Okpara University of Agriculture, Umudike, Nigeria; 10 Smithsonian Tropical Research Institute, Panama City, Panama; 11 Faculty of Chemistry and Earth Sciences, Institute of Geography, University of Heidelberg, Heidelberg, Germany; 12 Department of Geography ‑ Research Group for Earth Observation (rgeo), UNESCO Chair On World Heritage & Biosphere Reserve Observation and Education, University of Education Heidelberg, Heidelberg, Germany; 13 Geographical and Environmental Education Unit, Department of Social Science Education, University of Nigeria, Nsukka, Nigeria

**Keywords:** Ecology, Environmental sciences

## Abstract

This study aims to investigate soil organic carbon (SOC) and total nitrogen (TN) contents and stocks, CO_2_ emissions and selected soil properties in croplands, grazing lands, exclosures and forest lands of semi-arid Ethiopia. Sampling was done at 0–30, 30–60 and 60–90 cm soil depths and concentration and stocks of SOC, TN and selected soil properties were determined using standard routine laboratory procedures. There were variations in distribution of SOC and TN stock over 90 cm depth across land use types and locations, decreasing from topsoils to subsoil, with average values ranging from 48.68 Mg C ha^−1^ and 4.80 Mg N ha^−1^ in Hugumburda cropland to 303.53 Mg C ha^−1^ and 24.99 Mg N ha^−1^ in Desa’a forest respectively. Forest sequestered significant higher SOC and TN stock, decreasing with depth, compared with other land use types. In Desa'a and Hugumburda, the conversion of forest to cropland resulted in a total loss of SOC stock of 9.04 Mg C ha^−1^ and 2.05 Mg C ha^−1^, respectively, and an increase in CO_2_ emission of 33.16 Mg C ha^−1^ and 7.52 Mg C ha^−1^ yr^−1^, respectively. The establishment of 10 years (Geregera) and 6 years (Haikihelet) exclosures on degraded grazing land increased SOC stock by 13% and 37% respectively.

## Introduction

With about 2344 Gt of organic carbon (OC) sequestered in soils across the globe, the soil remains a vital carbon sink^1^ and is considered the principal terrestrial pool of OC^1,2^. Soil organic carbon (SOC) is stored both in top and subsoils. It is estimated that the global SOC stock is between 684–724 Pg at 30 cm depth and 1462–1548 Pg at 1 m depth^3^. At any given depth, SOC stocks depicts a balance between decomposition of organic matter and stabilization of assimilated carbon by soil microorganisms and this balance has the potential to change under different biophysical conditions (land use, vegetation, soil particle size distribution, soil pH, aggregate stability, precipitation)^4,5^. Furthermore, nitrogen can have control on SOC stocks by changing priming effects connected to N-mediated changes in soil microorganisms^6,7^. Biophysical gradients are useful to improve our understanding of how environmental and pedogenic factors affect soil processes and properties, because they contain long-term adaptive changes^8–10^. Therefore, understanding the distribution of SOC stocks in response to variations in soil physico-chemical properties, soil types and contrasting agro-ecosystems are essential to assess the coupling mechanism between terrestrial carbon–nitrogen cycling, environmental factors and climate change in tropical semi-arid environments.

Human activities tend to increase the size of SOC pool through series of land use including agricultural practices such as afforestation or conversion of cropland into grassland^11^, cover cropping, alley cropping, no-till and mixed cropping^1,12–15^. Land use change has been widely reported drivers of carbon dynamics in soils^16–18^. The SOC stocks in subsoil horizons originate from plant roots and root exudates, leaching and delivery of dissolved organic matter and bioturbation^19^. Depending on the nature of the soil, there is also a possibility of transport of clay-bound organic matter and translocation of particulate organic matter into subsoils^20^. More than 50% of the soil carbon stock is estimated to be found in the subsoil^21^, characterized by high mean residence time (MRT) and less prone to disturbance compared to topsoils^22^. Thus, in all terrestrial ecosystems including semi-arid and drylands, subsoils consist a major reservoir of organic C^19,21,23^. Clays alongside SOC are therefore involved in the formation of stable soil aggregates which minimizes SOC mineralization and depletion^24^. The importance of the aforementioned sources of SOC in subsoils is dependent on soil processes as well as land use and climatic parameters^20^. The SOC and nitrogen are interdependent. For example, an increase in CO_2_ will first lead to an increase in the net primary productivity of the soil ecosystem thereby leading to N immobilization in biomass, depleting soil N, increasing C:N ratio and reducing rates of mineralization^25^. Soil physicochemical properties including pH, CEC, particle size distribution and BD affect microbial diversity and abundance, and influence carbon and nitrogen cycling processes^26,27^. In recent times, however, land use and climate change have continued to accelerate the rate of soil aggregate breakdown thereby leading to loss of SOC and N^28^. Although the common knowledge at the moment is that land use change can result in loss of SOC and N^29,30^, human activities can lead to increased SOC and N stocks^1,11,13^, and SOC is generally lesser in semi-arid subsoils^6,31^, the interaction mechanism among MAP, CEC, clay content, soil pH and bulk density under landuse conversion gradient, and its impacts on depth distribution of SOC, N and CO_2_ emission in tropical semi-arid soils is still poorly understood^16,32^.

Ethiopia is a land-locked country with the second largest population in Africa and strategically positioned in the Horn of Africa. A low SOC content (< 1%) is concomitant with dryland soils^33^, leading to a high occurrence of degradation and an appreciable loss in SOC storage in Ethiopian soils^34–37^. Most studies on SOC stocks and N storage in northern Ethiopia^15,38,39^ focused only at 0–30 cm depth agricultural plough layer and without consideration of CO_2_ emissions. Therefore, there is a need for assessment of SOC and N distribution below the ploughing zone given that the impact of land use and climate change on SOC dynamics is not limited to the topsoil alone^40^.

The objectives of this study are:

(i) To quantify the distribution of SOC and TN concentrations and stocks and CO_2_ emissions within the profile to a depth of 90 cm in 30 cm increments in of four contrasting land use types (forest, exclosure, grazing land and cropland);
(ii) To quantify variations in selected soil properties (total nitrogen, pH, cation exchange capacity (CEC), particle size distribution and bulk density) in these land use types; and.
(iii) To identify biophysical controls (vegetation, soil properties, climate) on depth distribution of SOC, N storage and CO_2_ emission in tropical semi-arid soils of northern Ethiopia.

We hypothesized that (i) the impact of vegetation cover/land use on SOC and TN stocks and CO_2_ emissions in these soils is not limited to the topsoil layer, (ii) climate and land use will serve as possible indicators to modulate the magnitude of changes in SOC and TN stocks, (iii) SOC and TN concentrations and stocks decrease following conversion of forest to grazing land and cropland, but increase with establishment of exclosure on degraded grazing land.

## Materials and methods

### Description of the study area

**Figure 1:**
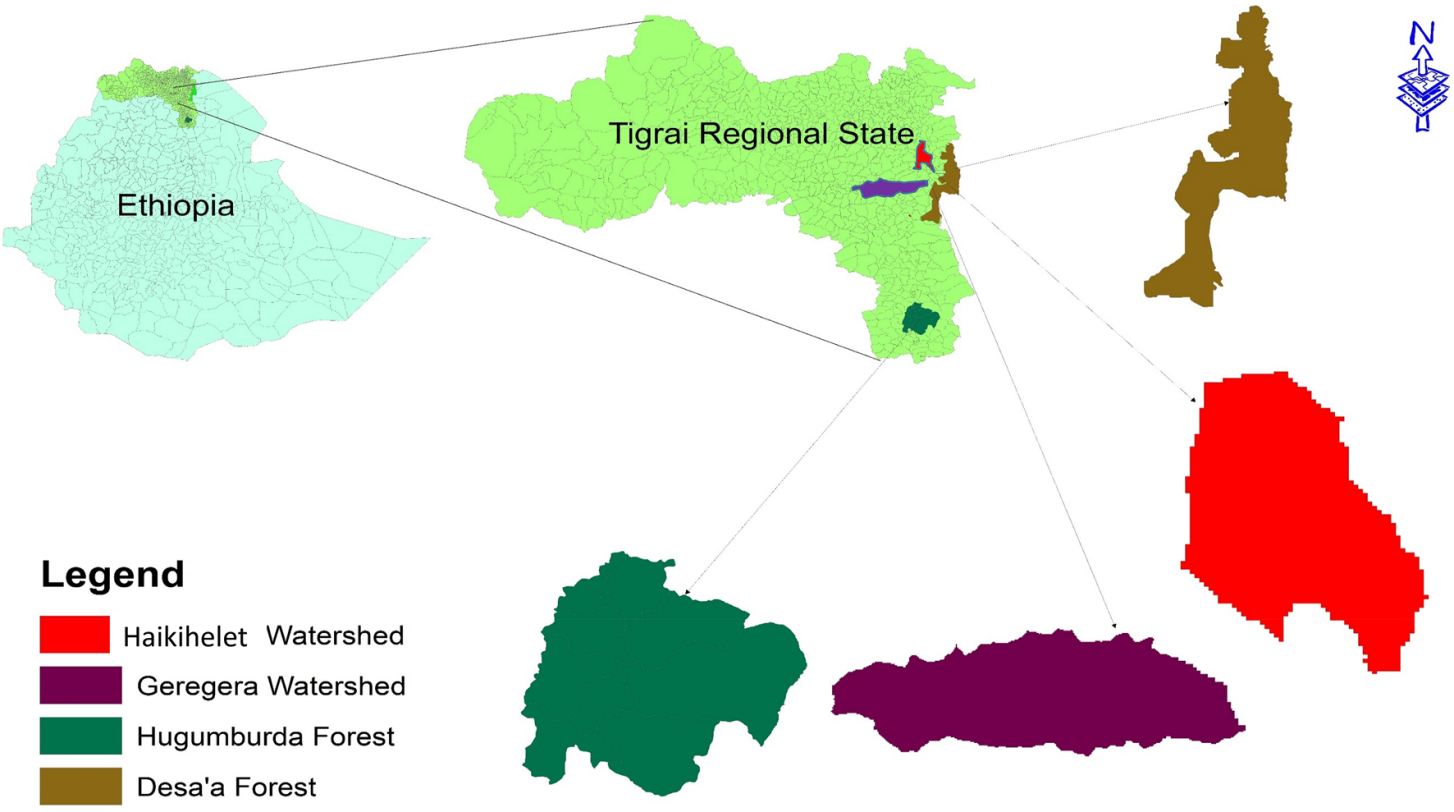
Location map of study area showing the study locations in semi-arid area of northern Ethiopia. *Source* Authors (ArcMap 10.4).

**Figure 2:**
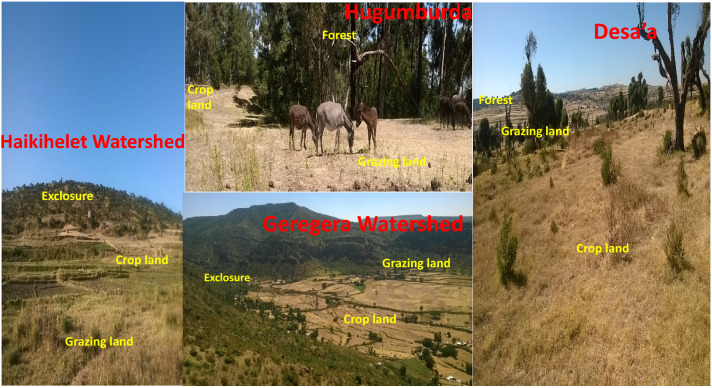
Pictorial view of all locations and their land use types (Courtesy of Okolo CC_December 2016).

Four locations (Fig. 1) with different land use types were chosen for this study: Hugumburda (natural forest, grazing land and cropland, 2494 m.a.s.l), Desa’a (natural forest, grazing land and cropland, 2433 m.a.s.l), Geregera watershed (exclosures, grazing land and cropland, 2180 m.a.s.l) and Haikihelet watershed (exclosures, grazing land and cropland, 2236 m.a.s.l), all in the semi-arid area of Tigray Regional State, northern Ethiopia (Fig. 2; Table 1). Exclosure refers to previously degraded grazing land, which has been fully conserved without any form of animal and human activity as a sustainable way of restoring land degradation through natural regeneration^41^. The age durations of the exclosures in the Haikihelet and Geregera watersheds are 6 and 10 years respectively^42^. Cambisols are the dominant soil types in Hugumburda, Haikihelet and Geregera, while Vertisols dominate in Desa’a^43^.

**Table 1:** Site description and meteorological data of study sites. The last crops at the point of sampling are the first two crops in each cropland and all sampled cropland soils were under rainfed cereal cultivation (Adapted from Okolo et al.^42,88^)

Location (Coordinates)	Land use	Mean annual temperature (°C)	Mean annual precipitation (mm)	Geological background	Soil type (WRB 2014)	Management type/dominant vegetation
Hugumburda 12° 40.441′ N 39° 32.05′	Forest	19	475	Tertiary basalt, alkali-alluvial basalt and tuff	Eutric Cambisols	Dry Afromontane forest with undergrowth and climbers, mainly *Juniperus procera Hochst. ex Endl., Maytenus obscura (A.Rich.) Cufod, Olea europaea ssp. cuspidata (Wall. Ex G.Don)*, *Pterolobium stellatum—Celtis Africana,* and *Cadia urpurea—Opuntia ficus-indica*. Traces of litter removal and tree cutting and carrying (for firewood) by the local dwellers
	Grazing land				Leptic Cambisols	Native grasses with remnants of Acacia and Tehag (shrub savannah) with sparsely grown patches of trees. Lightly and periodically grazed in a communal land. No fertilizer application and no cultivation
	Crop land				Vertic Cambisols	Rainfed cultivation of tef (*Eragrostis tef*), wheat (*Triticum aestivum*), *Hordeum vulgare* (barley), and Maize (*Zea mays*). NPK/manure application
Haikihelet 13° 39.3853′ N 39° 51.7760′ E	Exclosure	22	498	Limestone	Calcaric Cambisols	Undisturbed trees and shrubs, mainly *Acacia abyssinica* Hochst. ex Benth (‘Chea’) and Dodonia. Grazing, cultivation, and any form of human interference/activity is strictly prohibited. No cultivation/fertilization
	Grazing land				Calcaric Cambisols	Native grasses of ‘Rgihe’, ‘Gasa’, ‘Saeri’ and ‘Geza’ with Acacia and *Cynodon dactylon* (‘Tahay’). Intensively grazed in a communal land. Never cultivated. No fertilizer application
	Cropland				Calcaric Cambisols	Rainfed cultivation of *Hordeum vulgare* (barley), *Triticum aestivum* (wheat), and *Eragrostis teff* (*t*eff). Bunding, ploughing and NPK application/ manure
Desa’a 13° 38.879′ N 39° 46.282′ E	Forest	15	532	Enticho sandstone and Crystalline Precambrian Basement	Calcaric Cambisols	Dry Afromontane forest, mainly *Juniperus procera Hochst. ex Endl., Maytenus obscura (A.Rich.) Cufod, Olea europaea ssp. cuspidata (Wall. Ex G.Don) Cif., Cadia purpurea Ait., and Carissa edulis Vahl., Cadia purpuria* (G. Piccioli) Aiton and *Tarchonanthus camphoratus* L. Traces of litter removal and tree cutting and carrying (for firewood) by the local dwellers
	Grazing land				Pellic Vertisols	Native grasses with remnants of Juniperus and Olei Africana. Lightly and periodically grazed in a communal land. No cultivation and no fertilizer application
	Crop land				Calcic Vertisols	Rainfed cultivation of *Hordeum vulgare* (barley), *Triticum aestivum* (wheat) and *Eragrostis teff* (*t*eff)
Geregera 13° 45.118′ N 39° 43.602′ E	Exclosure	22	507	Adigrat and Enticho sandstones, with inclusion of Paleozoic sedimentary rocks and alluvial sediments	Gleyic Cambisols	*Juniperus procera* Hochst. ex Endl. (‘Tsihdi’), *Acacia abyssinica* Hochst. ex Benth (‘Chea’), *Olea European* subsp. *cuspidata* (‘Auli’e’*), Dodonea angustifolia* L. and *Eucalyptus globulus* Labill. (‘TsaedaBahrzaf’). *Euclea racemose* Murr. subsp. *schimperi* (A.DC.) F. Whit (‘Keleaw’) and *Beciumgrandiflorum* (‘Tebeb’). No cultivation/fertilization
	Grazing land				Gleyic Cambisols	Different species of ‘Susbania’ and diverse vegetation, including *Cynodon dactylon* (‘Tahay’) and *Hyperrhenia hirta*. (‘Goiti ebab’). Lightly and periodically grazed in a communal land. No cultivation/fertilization
	Crop land				Gleyic Cambisols	Rainfed and irrigation** cultivation of cereals: *Eragrostis teff* (*t*eff), *Hordeum vulgare* (barley), *Triticum aestivum* (wheat), *Sorghum bicolor* (sorghum) and *Zea mays* (maize); and pulses, example *Phaseolus vulgaris* (beans), *Lens culinaris* (lentil) and *Pisum sativum subsp. arvense* (field pea). Urea/DAP and animal manure application

**Irrigated croplands are among the major land use types in Geregera watershed as practiced by the local smallholder farmers but the irrigated croplands were not sampled in this study.

In the study area, the mean annual precipitation (MAP) between 1983 and 2016 is about 503 mm^44^. The rainy season peak period is in July/August and rescinds towards September. The estimated average temperature in the region is 18 °C, with significant variations with altitude. The study sites were classified as mid-altitude (1800–2200 m above sea level) and high altitude (> 2100 m above sea level) classes, based on the traditional indigenous agro-climate classification system in Ethiopia.

The most common crop rotation in the study area is wheat + barley + faba bean/field pea, while teff + maize can be switched after a few years. At the time of soil sampling, all cropland was under rainfed cereal crop cultivation. Sampling took place between November and December after harvest, when the soil conditions, particularly bulk density (since bulk density data are used to calculate carbon stocks) of tilled croplands had returned to their original pre-tillage state^45,46^.

### Soil sample collection and preparation

Soil sample collection was done in three soil layers: 0–30, 30–60 and 60–90 cm depth from forest, exclosures, grazing lands and croplands across the four study locations. Deep sampling to a depth of 90 cm with a sampling depth interval of 30 cm was used for the study as previous studies in the area were limited to only 0–30 cm depth. In addition, the deep sampling intervals were assumed to be consistent with the current standard soil depth of 30 cm as proposed for C accounting studies^47,48^. Systematic sampling was adopted for soil sample collection with transects established in each land use type. Soil samples were collected from three representative plots (50 × 50 m) on the established transect for each land use type. In all land use types, undisturbed samples were first collected using open-faced coring tube, before auger sampling from the same point. A hand-pushed auger was used in collecting the auger samples. Replicate plots within each land use type were approximately 400 m apart and the experimental plots were of the same lithology and management. Within each plot (replicate) in a land use type, auger samples were collected at each depth from four points of a soil profile pit (1.5 × 1 m), along the already established transect, giving four sampling positions per soil depth and twelve samples per land use type. The four soil auger samples collected from each depth were thoroughly mixed together to get one composite (representative) sample per depth in each plot. Nevertheless, in few land use types, sampling depth of 90 cm was not achieved as a result of depth-limiting occurrence of bedrocks at shallow depths. In general, a total number of 104 samples were obtained in all the four locations. Bulk density determination was carried out using the core samples. Bulk density samples were collected with the aid of core samplers, starting from the lowest soil depth (60–90 cm) to the topmost soil depth (0–30 cm). The auger samples were first air-dried, followed by manually removing the visible roots, twigs, debris and leaves, and finally sieving the soil using a 2.0 mm mesh screen. The < 2 mm samples were then subjected to further laboratory analysis.

### Laboratory analysis

Soil organic carbon content was determined using modified Walkley and Black wet oxidation method with H_2_SO_4_-K_2_Cr_2_O_7_ followed by residual titration with 1 N HCl^49^. Total nitrogen was determined by the modified macro Kjeldahl digestion method^50^. Soil pH was measured in soil–water (1:2.5) suspensions^51^. Cation exchange capacity (CEC) determination was by NH_4_OAC (pH 7) displacement method^52^. Bulk density was analyzed using core method^53^. Particle size distribution was determined by the hydrometer method^54^ using sodium hexametaphosphate as a dispersant. All measurements were taken in triplicates for improved accuracy.

### Calculations, estimations and statistical analyses

The SOC and TN stock in each land use type was calculated with the formula:

1 $$SOC\, or \, TN \left( {Mg \,C \,ha - 1} \right) = {\text{ concentration}}\frac{\% }{100} \times {\text{bulk}}\, {\text{density}}\left( {\frac{{{\text{Mg}}}}{{{\text{m}}3}}} \right) \times {\text{area }}\left( {{\text{ha}}} \right) \times {\text{soil}}\,{\text{depth}} \left( {\text{m}} \right)$$

where concentration (%) is the percentage concentration of carbon or nitrogen.

The total SOC and TN stocks to the depth of 90 cm across locations in each land type use was calculated by the summation of SOC and TN stocks in the 0–30, 30–60, and 60–90 cm soil depths^55^.

In Geregera and Haikihelet locations, using the grazing land as a baseline, SOC and TN stocks accumulation in exclosure within the same soil depth were obtained by calculating the difference in SOC and TN between exclosure and grazing land. Due to variation in periods of the different land use types, rate of SOC and TN stocks accumulation for each soil depth of the exclosure land use type was calculated by dividing the estimated accumulation values by the presumed period of exclosure establishment^56^.

The average age duration of Geregera and Haikihelet exclosures were given as 10 and 6 years respectively^42^. This simply implies the age duration in years since the exclosures were established from degraded grazing land. This information was based on the oral feedback from farmers (aged 60 years and above) in the study locations.

In Desa’a and Hugumburda locations, SOC and TN losses due to deforestation were estimated by subtracting the total SOC and TN stocks in forestland from its equivalent depth in grazing land or cropland. Thereafter, the calculated loss values were divided by the presumed period of years following land use conversion to get SOC and TN losses per year. The CO_2_ emission as a result of forest conversion to grazing land and cropland was then established on the basis of the underlying SOC and CO_2_ relationship as stated by^57^; which states that 1 Mg ha^−1^ increase in soil carbon signifies removal of 3.67 Mg of CO_2_ from the atmosphere. For the purpose of this study, we are focusing only on C lost as CO_2_ emission without consideration the C losses through erosion, and leaching in the form of dissolved organic C and sediment accumulation. From the obtained results of SOC and TN concentrations and stocks, the distribution trend was explained in the form of high, intermediate and low across the different land use types in all study locations for ease of comparison.

Considering that soil carbon quantity is specifically quantified in a particular soil depth for the purpose of C accounting and budgeting^47^, effects of land use on SOC and TN concentration and stock, CEC, pH, bulk density, was investigated, comparing them across same depth within site based on two-way analysis of variance (ANOVA). Log transformation of data was carried out before ANOVA whenever assumptions of normality and homogeneity of variances within a group were not obtained. Significant differences (p ≤ 0.05) were determined using Tukey’s honest significant difference (Tukey’s HSD) post hoc test. All the tests were carried using STATISTICA (Version 12.0, StatSoft GmbH, Hamburg, Germany). Factor analysis was performed using version 2014 of XLSTAT (Addinsoft, Paris, France).

## Results

### Soil organic carbon and total nitrogen (stocks and contents), and C:N ratios in top versus subsoils

**Figure 3:**
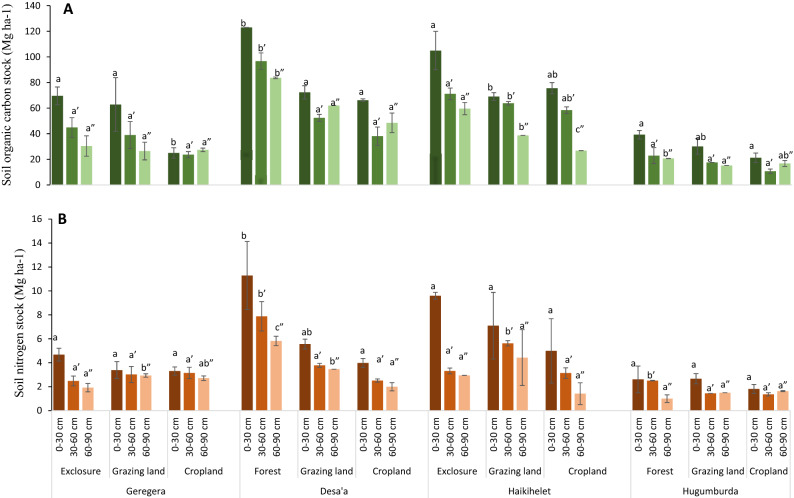
Soil organic carbon stock (**A**) and soil nitrogen stock (**B**) as influenced by land use and soil depth at Geregera, Haikihelet, Desa’a, and Hugumburda. Error bars represent the standard error of means. Letters above the error bars indicate significant differences (*p* ≤ 0.05) between land uses at 0–30 cm (a), 30–60 cm (a′) and 60–90 cm (a″).

Soil organic carbon and total nitrogen stocks and concentrations displayed a similar pattern—decreasing with soil depth among the land use types in all study locations (Fig. 3, Table 2). In Desa’a and Hugumburda, the C stocks per land use type is ranked as, forest (122.98 and 39.26 Mg C ha^−1^) > grazing land (72.31 and 30.03 Mg C ha^−1^) > cropland (66.19 and 21.22 Mg C ha^−1^) in the topsoil layer (0–30 cm depth) respectively (Fig. 3A). The trend of C stock distribution in top soil layer (0–30 cm depth) at Geregera is: exclosure (69 Mg C ha^−1^) > grazing land (63 Mg C ha^−1^) > cropland (24 Mg C ha^−1^), while the distribution trend in Haikihelet showed a similar ranking of: exclosure (105 Mg C ha^−1^) > grazing land (76 Mg C ha^−1^) > cropland (69 Mg C ha^−1^) (Fig. 3A). The TN stock showed significant (*p* ≤ 0.05) difference between land use types across locations, following a similar distribution pattern with SOC stock (Fig. 3B). Across all locations, SOC and TN stock decreased with soil depth, with high values in forest lands, medium in grazing lands and exclosures, and low in crop lands. The SOC and TN content (%) followed similar trend as the SOC and TN stock distribution (Table 2), decreasing with soil depth in all land use types across locations.

**Table 2:** Soil organic carbon and total nitrogen concentrations, C:N ratio, cation exchange capacity and pH under different land use types and soil depths in the study locations.

Location	Land use	Depth (cm)	Soil organic carbon (%)	Total nitrogen (%)	C:N	CEC (C mol kg^−1^)	pH (H_2_O)
Hugumburda	Forest	0–30	1.16 ± 0.11c	0.07 ± 0.00a	15.66 ± 0.05a	58.12 ± 0.12c	7.90 ± 0.06b
		30–60	0.66 + 0.17c′	0.08 ± 0.03a′	8.79 ± 0.10a′	56.52 ± 0.14b′	7.75 ± 0.02b′
		60–90	0.53 ± 0.03b″	0.03 ± 0.01a″	20.00 ± 0.02b″	50.95 ± 0.17a″	7.85 ± 0.03c″
	Grazing land	0–30	0.85 ± 0.20ab	0.08 ± 0.01a	11.31 ± 0.11a	34.39 ± 0.00a	7.55 ± 0.03a
		30–60	0.40 + 0.00ab′	0.03 ± 0.00a′	12.21 ± 0.00a′	28.12 ± 3.18a′	7.60 ± 0.00a′
		60–90	0.40 ± 0.01a″	0.04 ± 0.00a″	10.10 ± 0.00a″	19.02 ± 0.00b″	7.60 ± 0.00a″
	Crop land	0–30	0.50 ± 0.06a	0.05 ± 0.01a	11.21 ± 0.04a	45.27 ± 2.55b	8.05 ± 0.02c
		30–60	0.25 + 0.03a′	0.03 ± 0.00a′	7.66 ± 0.02a′	32.65 ± 2.87a′	7.90 ± 0.00c′
		60–90	0.40 ± 0.00a″	0.04 ± 0.01a″	9.86 ± 0.01a″	39.12 ± 6.29a″	7.75 ± 0.03b″
Haikihelet	Exclosure	0–30	2.83 ± 0.50a	0.20 ± 0.09a	14.15 ± 0.29a	30.51 ± 2.24b	7.90 ± 0.00a
		30–60	1.74 + 0.07a′	0.14 ± 0.00b′	12.42 ± 0.04b′	19.55 ± 3.78a′	7.96 ± 0.03a′
		60–90	1.46 ± 0.01c″	0.11 ± 0.06a′′	13.27 ± 0.01a″	14.63 ± 0.00a″	8.00 ± 0.00a″
	Grazing land	0–30	2.09 ± 0.13a	0.14 ± 0.01a	15.13 ± 0.07a	15.12 ± 6.05a	7.30 ± 0.00b
		30–60	1.54 + 0.14a′	0.08 ± 0.01a′	18.55 ± 0.07a′	5.25 ± 0.85b′	7.55 ± 0.03b′
		60–90	0.59 ± 0.0a″	0.03 ± 0.00a′′	18.90 ± 0.00b″	3.60 ± 1.36b″	7.80 ± 0.00a″
	Crop land	0–30	1.83 ± 0.03a	0.26 ± 0.08a	7.04 ± 0.06b	24.95 ± 0.05ab	7.95 ± 0.03a
		30–60	1.54 + 0.03a′	0.08 ± 0.00a′	19.25 ± 0.01b′	17.28 ± 3.75a′	7.95 ± 0.02a′
		60–90	1.00 ± 0.00b″	0.07 ± 0.01a″	14.28 ± 0.03a″	17.12 ± 0.00a″	8.10 ± 0.00a″
Desa′a	Forest	0–30	3.88 ± 0.00c	0.36 ± 0.01b	10.88 ± 0.01a	25.83 ± 2.99a	7.40 ± 0.06b
		30–60	3.04 + 2.00c′	0.25 ± 0.09b′	12.27 ± 0.15a′	23.40 ± 1.98a′	7.65 ± 0.03b′
		60–90	2.44 ± 0.04b″	0.17 ± 0.03c″	14.36 ± 0.04a″	19.63 ± 3.12a″	7.75 ± 0.03c″
	Grazing land	0–30	2.09 ± 0.04b	0.16 ± 0.00a	13.01 ± 0.02a	32.28 ± 0.99a	7.25 ± 0.03a
		30–60	1.55 + 0.01b′	0.11 ± 0.01a′	13.73 ± 0.01a′	28.27 ± 0.00a′	7.20 ± 0.00a′
		60–90	1.47 ± 0.00a″	0.08 ± 0.00b″	17.91 ± 0.00a″	24.47 ± 1.98a″	7.30 ± 0.00a″
	Crop land	0–30	1.80 ± 0.02a	0.11 ± 0.01a	16.63 ± 0.01a	28.97 ± 0.43a	7.55 ± 0.02c
		30–60	0.89 + 0.19a′	0.06 ± 0.00a′	14.83 ± 0.10a′	26.63 ± 2.58a′	7.75 ± 0.02c′
		60–90	1.14 ± 0.20a″	0.05 ± 0.01a″	22.80 ± 0.10a″	21.81 ± 2.02a″	7.45 ± 0.03b″
Geregera	Exclosure	0–30	1.48 ± 0.57ab	0.08 ± 0.02a	18.86 ± 0.29b	18.52 ± 3.06a	7.55 ± 0.03a
		30–60	0.92 + 0.21a′	0.07 ± 0.01a′	12.84 ± 0.11b′	19.84 ± 1.58a′	7.50 ± 0.00a′
		60–90	0.62 ± 0.18a″	0.07 ± 0.00a″	9.11 ± 0.09a″	8.35 ± 4.09a″	7.50 ± 0.00a″
	Grazing land	0–30	1.88 ± 0.10b	0.13 ± 0.01a	14.89 ± 0.05ab	24.59 ± 6.57a	7.50 ± 0.00a
		30–60	1.19 + 0.22a′	0.07 ± 0.01a′	18.09 ± 0.11c′	24.52 ± 8.84a′	7.45 ± 0.03a′
		60–90	0.71 ± 0.19a″	0.05 ± 0.01a″	15.87 ± 0.10b″	19.68 ± 8.99a″	7.35 ± 0.03b″
	Crop land	0–30	0.58 ± 0.08a	0.08 ± 0.01a	7.25 ± 0.05a	14.64 ± 0.66a	8.10 ± 0.00b
		30–60	0.57 + 0.08a′	0.08 ± 0.01a′	7.13 ± 0.04a′	10.94 ± 0.03a′	7.80 ± 0.06b′
		60–90	0.61 ± 0.05a″	0.06 ± 0.01a″	10.16 ± 0.03a″	6.44 ± 1.53a″	7.55 ± 0.03a″

± Mean followed by standard errors. Letters after the standard errors indicate significant differences (P < 0.05) between land uses at 0–30 cm (a), 30–60 cm (a′) and 60–90 cm (a″).

There was no clear distribution pattern of C:N ratio with depth under different land use types. The soil C:N ratios were high in forest and low in cropland across locations, except for the cropland in Desa’a which recorded higher C:N ratio than forest (Table 2). Across all locations, the soil C:N ratios ranged between 22.80 and 7.04.

### Accumulation and loss of SOC and TN stock

With exclosure establishment, high SOC stock accumulation in Geregera (16.57 Mg C ha^−1^) and Haikihelet (64.20 Mg C ha^−1^) was observed (Table 3). The high SOC accumulation also accounted for a high SOC accumulation rate of 6.88 Mg C ha^−1^ yr^−1^ in Geregera and 10.7 Mg C ha^−1^ in Haikihelet. The TN accumulation and rate of TN accumulation for Geregera and Haikihelet were 0.18 Mg N ha^−1^ and 0.02 Mg TN ha^−1^ yr^−1^ and 1.29 Mg N ha^−1^ and 0.22 Mg TN ha^−1^ yr^−1^ respectively (Table 3). The estimated total loss of SOC resulting from forest conversion to cropland led to SOC loss of 9.04 Mg ha^−1^ yr^−1^ and 2.05 Mg ha^−1^ yr^−1^ in Desa’a and Hugumburda respectively. Also, conversion of forest to grazing land accounted for a total SOC loss of 3.50 Mg ha^−1^ yr^−1^ and 0.61 Mg ha^−1^ yr^−1^ in Desa’a and Hugumburda, respectively (Table 4). Similarly, forest conversion to cropland gave rise to emission of 33.16 Mg ha^−1^ yr^−1^ CO_2_ and 7.52 Mg ha^−1^ yr^−1^ CO_2_ in Desa’a and Hugumburda, respectively (Table 4). More so, the conversion of forest to grazing land accounted for an emission of 12.84 Mg ha^−1^ yr^−1^ CO_2_ and 2.21 Mg ha^−1^ yr^−1^ CO_2_ in Desa’a and Hugumburda respectively.

**Table 3:** Magnitude and rate of soil organic carbon (SOC) and total nitrogen (TN) stocks accumulation in exclosures at 0–90 cm depth in Geregera and Haikihelet [grazing land as baseline].

Location	Land use	Assumed duration since conversion (Year)	SOC stock (Mg C ha^−1^)	SOC Accumulation (Stock—Cropland) (Mg C ha^−1^)	Rate of SOC Accumulation (Mg C ha^−1^ yr^−1^)	TN stock (Mg N ha^−1^)	TN Accumulation (Stock—Cropland) (Mg N ha^−1^)	Rate of TN Accumulation (Mg N ha^−1^ yr^−1^)
Geregera	Grazing exclosure	–	128.28			9.17		
		10	144.85	16.57	1.66	9.35	0.18	0.02
Haikihelet	Grazing exclosure	–	171.48			15.86		
		6	235.68	64.2	10.7	17.15	1.29	0.22

**Table 4:** Soil carbon and nitrogen loss and calculated potential carbon dioxide emission related to a change in land use (conversion from forest [baseline] to grazing and cropland) in Desa’a and Hugumburda.

Location	Land use	Depth (cm)	SOC loss (Mg C ha^−1^ yr^−1^)	N loss (Mg N ha^−1^ yr^−1^)	CO_2_ loss (Mg C ha^−1^ yr^−1^)
Desa’a	Grazing land	0–30	1.52	0.17	5.58
		30–60	1.33	0.12	4.88
		60–90	0.65	0.07	2.38
		Total	3.50	0.36	12.84
	Crop land	0–30	3.41	0.44	12.50
		30–60	3.52	0.32	12.92
		60–90	2.11	0.23	7.74
		Total	9.04	0.99	33.16
Hugumburda	Grazing land	0–30	0.28	0.00	1.02
		30–60	0.16	0.03	0.58
		60–90	0.17	0.00	0.61
		Total	0.61	0.03	2.21
	Crop land	0–30	1.08	0.05	3.97
		30–60	0.73	0.07	2.69
		60–90	0.24	0.00	0.86
		Total	2.05	0.12	7.52

Regarding TN loss, forest conversion to cropland accounted for a TN loss of 0.99 Mg ha^−1^ yr^−1^ and 0.12 Mg ha^−1^ yr^−1^ in Desa’a and Hugumburda respectively. More so, conversion of forest to grazing land accounted for very low TN loss in Desa’a and Hugumburda (Table 4).

In Desa’a and Hugumburda, conversion of forest to grazing land and cropland accounted for huge SOC stock depletion amounting to 30 to 50% of the SOC in the topsoil layer. Forest conversion to grazing land in Desa’a resulted to a reduction in total SOC and TN stock of 3.50 Mg C ha^−1^ yr^−1^ and 0.36 Mg N ha^−1^ yr^−1^ in grazing land, and 9.04 Mg C ha^−1^ yr^−1^ and 0.99 Mg N ha^−1^ yr^−1^ in cropland respectively. Hugumburda followed the same pattern of SOC and TN stock loss, though with less magnitude accounting for estimated total SOC and TN stock loss of 0.61 Mg C ha^−1^ yr^−1^ and 0.03 Mg N ha^−1^ yr^−1^ in grazing land, and 2.05 Mg C ha^−1^ yr^−1^ and 0.12 Mg N ha^−1^ yr^−1^ in cropland respectively (Table 4).

### Soil physicochemical properties

The CEC was consistently higher in the upper (0–30 cm) than the lower (30–60 and 60–90 cm) soil layers under all land use types across locations. The highest CEC value of 58.12 cmol kg^−1^ was recorded in the 0–30 cm forest soil at Hugumburda with the least CEC value obtained at 60–90 cm grazing land at Haikihelet (3.60 cmol kg^−1^) (Table 2).

In Vertisols of Desa’a location, no land use effect on CEC was observed, whereas in all other locations, predominated by Cambisols, croplands displayed higher CEC values more than grazing land, with the exception of Geregera. There was an observed decrease in CEC content across soil depths in all land use types in studied locations.

Land use types and soil depth affected the pH value of the soils, though with narrow margin across locations. The highest soil pH of 8.10 was recorded in Geregera cropland 0–30 and Haikihelet cropland 60–90 while the least soil pH value of 7.2 was recorded in subsoil (30–60 cm) of Desa’a grazing land (Table 2). Generally, the soil pH was in the slightly alkaline range in the study area (Table 2).

Particle size distribution of the soils showed significant (*p* ≤ 0.05) variations across depths under different land use types (Table 5) with an exception in Hugumburda where total sand and silt fractions showed no significant difference (*p* ≥ 0.05) across depths among land use types. Predominance of total sand fraction in Cambisols of Hugumburda and Geregera in different land use types was observed. In Cambisols of Haikihelet, total sand fraction was dominant in exclosure, while clay and silt fractions were dominant in grazing land and cropland respectively. In Vertisols of Desa’a, silt fraction was dominant in forestland while clay fraction was dominant in grazing land and cropland. Clay fraction increased with depth in most land use types across locations except in Hugumburda (forest and grazing land), Haikihelet (forest, grazing land and cropland) and Desa’a (forest) (Table 5).

**Table 5:** Soil physical properties under different land uses and soil depths in the study locations.

Location	Land use	Depth (cm)	Total sand (%)	Silt (%)	Clay (%)	Textural class (USDA)	Bulk density (Mg/m^3^)
Hugumburda	Forest	0–30	46.00 ± 0.57a	43.00 ± 2.30a	11.00 ± 1.73a	Loam	1.13 ± 0.01a
		30–60	48.00 + 4.03a′	40.00 ± 4.00a′	12.00 ± 0.00a′	Loam	1.15 ± 0.01b′
		60–90	59.00 ± 1.15a″	30.00 ± 0.58a″	11.00 ± 0.58a″	Loam	1.31 ± 0.07a″
	Grazing land	0–30	44.00 ± 5.20a	39.00 ± 5.77a	17.00 ± 0.57a	Loam	1.19 ± 0.04a
		30–60	57.00 + 0.00a′	27.00 ± 0.00a′	16.00 ± 0.00a′	Sandy Loam	1.45 ± 0.00a′
		60–90	49.00 ± 0.00a″	39.00 ± 0.00a″	12.00 ± 0.00a″	Sandy Loam	1.26 ± 0.00a″
	Crop land	0–30	46.00 ± 1.73a	42.00 ± 4.04a	12.00 ± 2.31a	Loam	1.38 ± 0.08b
		30–60	49.00 + 5.77a′	37.00 ± 8.08a′	14.00 ± 2.20a′	Loam	1.44 ± 0.07a′
		60–90	48.00 ± 5.19a″	35.00 ± 6.93a″	17.00 ± 1.73b″	Loam	1.42 ± 0.18a″
Haikihelet	Exclosure	0–30	45.00 ± 4.62b	32.00 ± 0.58a	23.00 ± 5.19a	Loam	1.21 ± 0.01a
		30–60	39.00 + 6.93b′	35.00 ± 1.15a′	26.00 ± 8.08a′	Clay Loam	1.28 ± 0.06a′
		60–90	57.00 ± 0.00b″	33.00 ± 0.00a″	10.00 ± 0.00b″	Sandy Loam	1.53 ± 0.00b″
	Grazing land	0–30	18.00 ± 5.19a	30.00 ± 0.58a	52.00 ± 5.77b	Clay	1.25 ± 0.05a
		30–60	13.00 + 4.62a′	30.00 ± 4.04a′	57.00 ± 8.66b′	Clay	1.37 ± 0.14a′
		60–90	20.00 ± 7.51a″	27.00 ± 4.62a″	53.00 ± 12.12b″	Clay	1.36 ± .10ab″
	Crop land	0–30	23.00 ± 3.46a	45.00 ± 2.31b	32.00 ± 5.77a	Clay Loam	1.26 ± 0.08a
		30–60	14.00 + 0.58a′	57.00 ± 4.62b′	29.00 ± 4.04a′	Silty clay loam	1.39 ± 0.05a′
		60–90	19.00 ± 0.00a″	49.00 ± 0.00b″	32.00 ± 0.00ab″	Silty clay loam	1.29 ± 0.00a″
Desa’a	Forest	0–30	30.00 ± 6.35a	40.00 ± 5.19b	30.00 ± 1.15a	Clay loam	1.06 ± 0.00a
		30–60	28.00 + 6.35b′	43.00 ± 9.24b′	29.00 ± 2.89b′	Clay loam	1.06 ± 0.00a′
		60–90	22.00 ± 5.19b″	47.00 ± 5.77b″	31.00 ± 0.58b″	Silty clay loam	1.14 ± 0.01b″
	Grazing land	0–30	30.00 ± 6.35a	32.00 ± 2.89ab	38.00 ± 9.24a	Clay loam	1.15 ± 0.06ab
		30–60	12.00 + 1.73a′	26.00 ± 2.89ab′	62.00 ± 4.62a′	Clay	1.13 ± 0.05a′
		60–90	9.00 ± 0.00ab″	19.00 ± 0.00a″	72.00 ± 0.00a″	Clay	1.41 ± 0.00a″
	Crop land	0–30	9.00 ± 00.00b	23.00 ± 0.00a	68.00 ± 0.00b	Clay	1.22 ± 0.01b
		30–60	8.00 + 0.58a′	20.00 ± 1.73a′	72.00 ± 2.31a′	Clay	1.44 ± 0.05b′
		60–90	7.00 ± 1.15a″	23.00 ± 2.31a″	70.00 ± 3.46a″	Clay	1.42 ± 0.03a″
Geregera	Exclosure	0–30	49.00 ± 1.15a	28.00 ± 0.58a	23.00 ± 1.73a	Sandy clay loam	1.23 ± 0.06a
		30–60	48.00 + 5.19a′	25.00 ± 8.08a′	27.00 ± 2.89a′	Sandy clay loam	1.26 ± 0.01a′
		60–90	56.00 ± 6.35a″	19.00 ± 6.93a″	25.00 ± 0.58a″	Sandy clay loam	1.41 ± 0.01a″
	Grazing land	0–30	40.00 ± 4.04b	26.00 ± 0.58a	34.00 ± 3.46b	Clay loam	1.49 ± 0.10b
		30–60	47.00 + 16.17a′	13.00 ± 3.46a′	40.00 ± 12.70a′	Sandy clay	1.38 ± 0.01a′
		60–90	49.00 ± 17.32a″	12.00 ± 4.04a″	39.00 ± 12.28a″	Sandy clay	1.43 ± 0.03a″
	Crop land	0–30	51.00 ± 1.15a	22.00 ± 0.58b	27.00 ± 0.58ab	Sandy clay loam	1.44 ± 0.04ab
		30–60	45.00 + 6.93a′	19.00 ± 3.46a′	36.00 ± 3.46a′	Sandy clay loam	1.39 ± 0.05a′
		60–90	39.00 ± 6.92a″	19.00 ± 4.62a″	42.00 ± 2.31a″	Clay	1.51 ± 0.04a″

± Mean followed by standard errors. Letters after the standard errors indicate significant differences (*p* < 0.05) between land uses at 0–30 cm (a), 30–60 cm (a′) and 60–90 cm (a″).

Bulk density (BD) differed significantly (*p* ≤ 0.05) with depths across different land use types in the studied locations. Similarity existed between topsoil BD of grazing land and cropland. Across locations, the highest BD value of 1.53 Mg/m^3^ was recorded in 60–90 cm of Haikihelet exclosure while the lowest BD value of 1.06 Mg/m^3^ was recorded in both 0–30 and 30–60 cm depth in Desa’a forestland. Significant increase in BD with depth was observed across locations (Table 5).

### Factor analysis across locations

**Figure 4:**
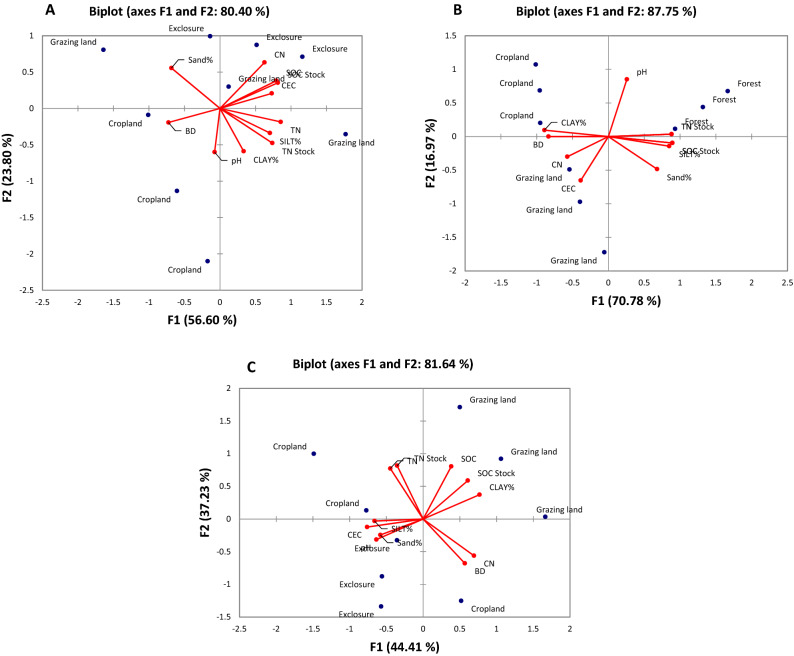
Biplots of (**A**) Geregera (**B**) Desa’a, and (**C**) Haikihelet locations as influenced by land use. Soil physico-chemical characteristics: BD = bulk density, Soil texture (sand%, silt% and clay%), SOC = soil organic carbon, TN = total nitrogen, CEC = cation exchange capacity, CN = C:N ratio. The red arrow denotes the direction of the high weighting of soil physico-chemical characteristics in the first (FA-1) and second factor (FA-2).

In Geregera, the first factor axis (F1) of the biplots relates to plots gradient from grazing land to cropland, however, there is similarity between the plots of grazing land and forest land. Most of the studied soil properties (SOC, TN, C:N ratio, CEC, SOC and TN stock, pH, % silt and % clay) were higher in exclosure and grazing land compared to cropland (Fig. 4A). Notably, cropland soils are characterized by loam, sandy loam, silty clay loam and sandy clay loam texture for Hugumburda, Haikihelet, Desa’a and Geregera respectively (Table 2), in addition to low SOC content as observed in the second factor axis (F2) which explained 23.80% of the total variance. In general, this relates to a particular gradient of low SOC (cropland plots) to high SOC (exclosure plots) (Fig. 4A).

In Desa’a, the first factor axis (F1) of the biplots followed particular pattern modulated by percent clay distribution. Cropland has higher percent clay content, followed by grazing land, with forest land recording the least percent clay content (Fig. 4B). The second factor axis (F2) of the biplots indicates a pattern of inclination from forest to cropland plots. However, a clear pattern was observed, starting from forest with high SOC and high sand fraction to cropland with low SOC and low sand fraction (Fig. 4B).

The biplots in Haikihelet indicated that the first factor axis (F1) exhibited a clear pattern of inclination from exclosure to cropland plots. The second factor axis (F2) followed a different pattern of increment from cropland to grazing land, corresponding to a gradient of low to high SOC pool. The grazing land is characterized by high SOC content, SOC stock and high clay content. (Fig. 4C).

**Figure 5:**
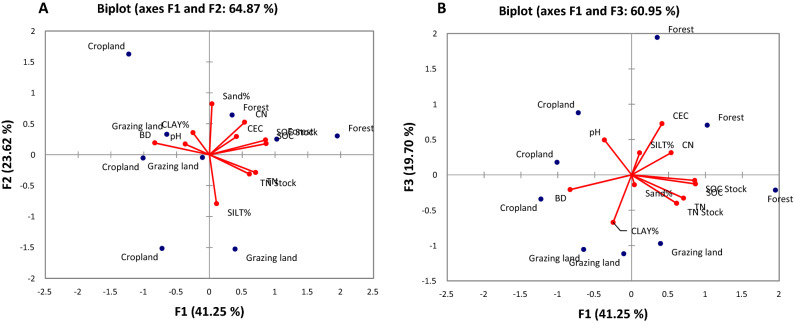
Biplots of Hugumburda depending on landuse indicating (**A**) Factor axis F1 and F2, and (**B**) Factor axis F1 and F3, as influenced by land use. Soil physico-chemical characteristics: BD = bulk density, Soil texture (sand%, silt% and clay%), SOC = soil organic carbon, TN = total nitrogen, CEC = cation exchange capacity, CN = C:N ratio. The red arrow denotes the direction of the high weighting of soil physico-chemical characteristics in the first (FA-1) and second factor (FA-2).

It was observed that the biplots in Hugumburda both for the first factor axis (F1) and second factor axis (F2) of the biplots explained approximately 65% of the variances in the components (Fig. 5A), thus necessecitating to incorporate third factor axis (F3) (Fig. 5B). The first factor axis (F1) explained 41.25% of the total variance in forest and partly in grazing land, consisiting of variables indicative of soil nutrient availability (SOC, TN, SOC stock, TN stock, CEC, and C:N ratio) (Fig. 5A). The second factor axis (F2) which explained 23.62% of the total variance, was characterized by % clay, BD and pH in cropland and grazing land. The third factor axis (F3) explained 19.70% of total variation, and was characterized by pH and BD in grazing land and cropland and so, reflects the impact of anthropogenic activities (Fig. 5B).

## Discussion

Significant difference in SOC and TN stocks was observed among various land use types across depths, with clear differences in distribution trend across locations. The high SOC and TN concentration recorded in topsoil (0–30 cm) of forest and exclosure could be as a result of minimal disturbance in these ecosystems, litter accumulation from trees and shrubs, below ground litter and high biomass cover^58–60^. In addition, SOC and TN distribution at lower depth in most land use types were usually low at 60–90 cm depth, thus indicating that effect of land-use was mainly limited to the upper soil layers. This did not support our first hypothesis which states that the impact of land use is not limited to the topsoil. The significantly lower concentrations of SOC and TN especially in cropland soils across depths could be attributed to unsustainable farm practices like total harvesting without residue retention thus exposing the soil to incidences of soil and water erosion, residue burning and intensive tillage operations which exacerbates decomposition and high rate of SOM oxidation due to continuous cultivation^61–63^. However, with good management practices in croplands, C retention and stabilization can be enhanced.

A possible attribution of high SOC contents at Haikihelet and Desa’a is the high clay content recorded in these locations (See Table 5). In this study, both SOC and clay contents were found to be higher in Vertisols (clay dominated) than Cambisols (sandy loam dominated). This finding is in line with works of^64^ and^65^ who reported that clay textured soils had higher SOC content in studies assessing distribution of SOC levels and structural indices under contrasting land use types in southeastern Nigeria.

The forest sequestered more SOC stock than other studied land use systems, with high sequestration in topsoils. This is in line with the report of^16^ that forest soils are great C pools of terrestrial ecosystems in the global C cycle. This implies a high risk of CO_2_ release from these forest topsoils if they are eventually deforested or converted to cropland. The low SOC stocks in the cropland are attributable to the total harvest, tillage activities in addition to leaching and erosion losses and reduced organic material going back to the soil, soil and water erosion leading to loss of SOM, regular tillage and cropping activities accounting for high oxidation rates of SOM, burning of crop residues^19,61,66^.

Our results show that C:N ratio was affected by land use types, but there was no definite distribution trend. This suggests that the sole use of C:N ratio as a SOM quality indicator is limited and quite misleading^67^. The lowest C:N ratio in 30–60 cm depth in cropland soil of Hugumburda (7.66) corresponds to a very low SOC content of 0.25% (Table 2). The ratio was much narrower in croplands (with the exception of Desa’a cropland) than other land use types, which is an indication of high mineralization and oxidation rate in cropland soils. Decline in C:N ratio with soil depth is evident in most agricultural soils^68^. Interpretation of changes in C:N ratio due to in land use changes or management practices is complex and has been suggested to be better treated separately from SOC and TN concentrations and stocks, and on a case by case basis for clear understanding^67^.

At Geregera and Haikihelet, taking grazing land as the baseline, increase in SOC and TN stock accumulation as a result of exclosure establishment was recorded. The observed improvement in SOC and TN stock in 6- and 10-year-old exclosure in Haikihelet and Geregera respectively is attributed to increase in organic inputs due to vegetation restoration and restriction of animal grazing on exclosures. This firmly supports section of our third hypothesis which states that exclosure establishment on degraded ecosystems results in SOC restoration. The implication of our result is that long age duration of exclosures may not necessarily result to remarkable replenishment of soil nutrients on previously degraded grazing land in compared to short-term exclosures. Site-specific characteristics and micro-climatic conditions across locations might have contributed to these variations in SOC accumulation rates.

Most of the SOC and TN stocks losses were in the 0–30 cm topsoil layer across land use types (Table 4). Similar trend in SOC loss has been previously reported by^34^ who indicated that forest conversion to crop land, open grazing, and plantation accounted for an estimated decline in SOC stock in the topsoil layer amounting to 0–63% in cropland, 0–23% in open grazing land, and 17–83% in plantation. This confirms a section of our third hypothesis which states that SOC concentrations and stocks decrease after conversion of natural forests to cultivated lands. In the dry Afromontane remnant pristine forests in northwest Ethiopia, huge reduction in SOC stock of up to 87% and 50% with the conversion of forest to cropland at Katassi and Gelawdios sites respectively was reported by^59^. This portends a huge threat to global warming in the face of climate change.

Overall, with forest conversion to cropland and grazing land, the estimated CO_2_ emission as obtained in this study is huge and capable of contributing to atmospheric greenhouse gas effect. The CO_2_ emissions decreased with soil depth with higher emissions in cropland compared to grazing land soils. Notably, CO_2_ fluxes decreases appreciably with depth though not significantly contributing to surface fluxes^69,70^. This implies that any form of subsoil disturbance could affect the deep subsoil CO_2_ reservoir. Thus, mobility of CO_2_ in the subsoil to the surface soil is impaired and may be entrapped in soil pores and solution if undisturbed, or used by subsoil autotrophs^19^. In cropland soils with high CO_2_ emissions, these CO_2_ loss effects can be compensated by the accrual of deep root C inputs from deep-rooting crops. Recent studies of deep-rooted perennial grasses planted in C-poor soils reported no effect of these crops on surface CO_2_ fluxes in different soil types^71,72^. Nevertheless, the value of SOC loss in our study indicates that loss of SOC may not only be as a result of CO_2_ emission to the atmosphere^73^ but can as well be lost due to leaching in the form of dissolved organic carbon, erosion and sediment accumulation, which were not considered in this study. Thus, the actual fate of this SOC loss across landscape in semi-arid area of northern Ethiopia is still not well-known and there is need for further detailed investigation.

**Figure 6:**
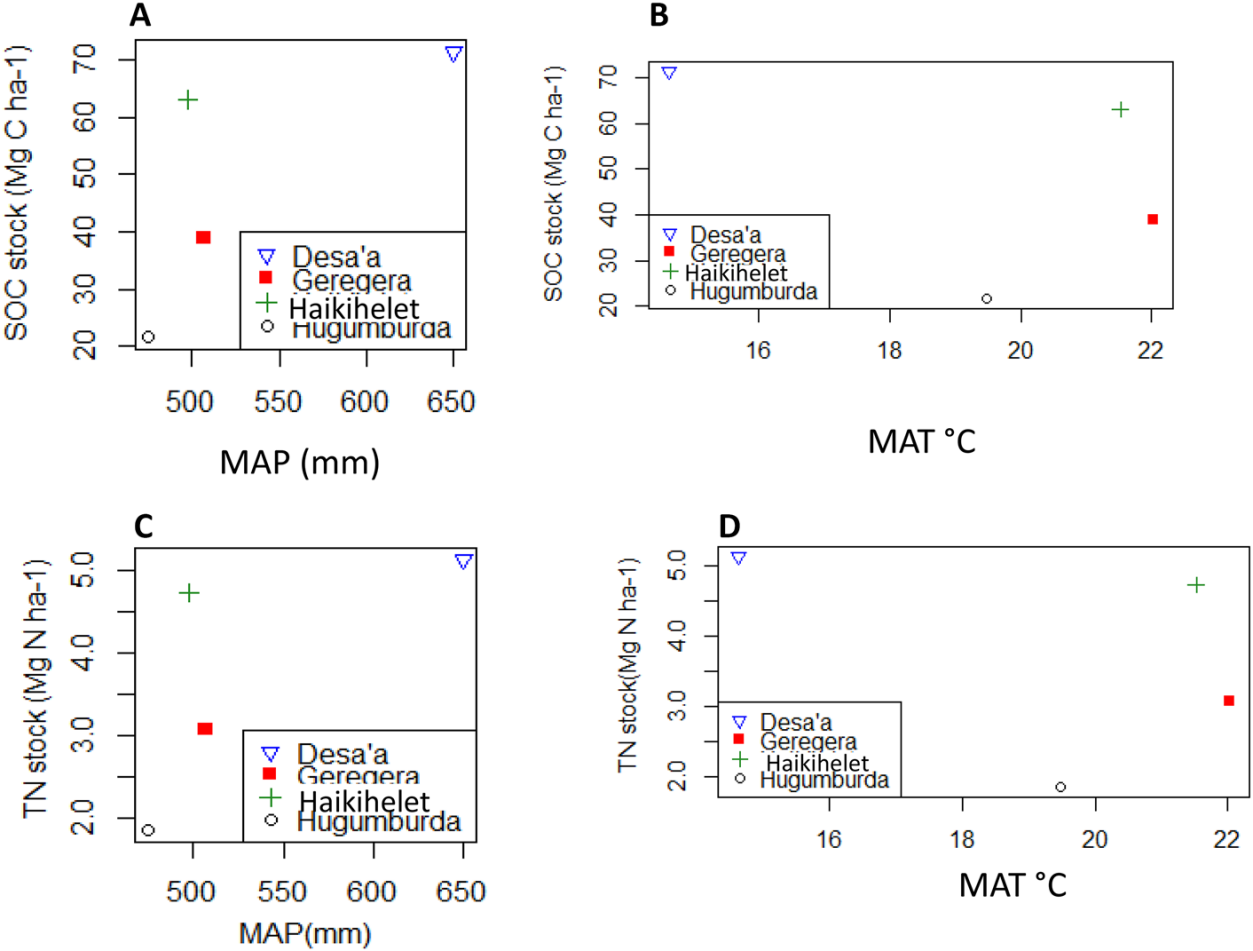
Linear relationship between soil carbon stock (Mg C/ha) and (**A**) mean annual precipitation (MAP); (**B**) and mean annual temperature (MAT); and total nitrogen stock (Mg N/ha) and (**C**) mean annual precipitation (MAP); (**D**) and mean annual temperature (MAT); (*p* ≤ 0.05, n = 16).

**Figure 7:**
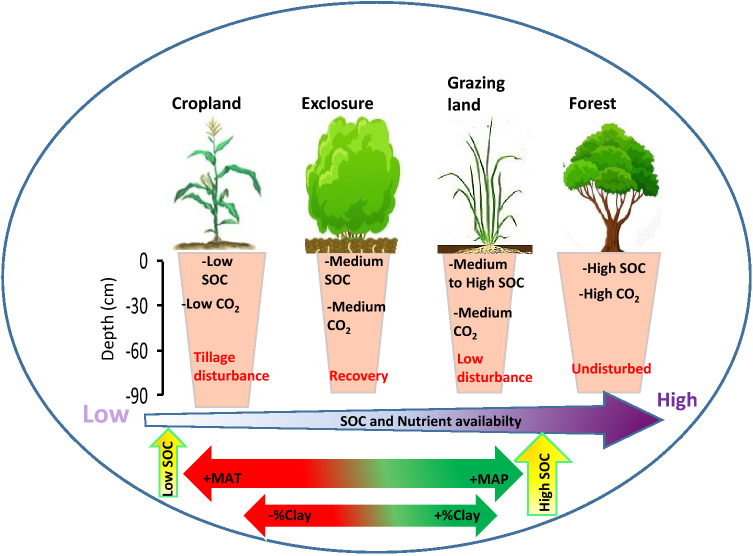
Conceptual diagram summarizing factors and mechanisms driving SOC distribution under different land use types in semi-arid area of northern Ethiopia. The nutrient availability arrow illustrates the concentration pathway and distribution of SOC and TN contents across the land use types. The double-headed arrows indicates the direction of both MAT and %silt in modulating SOC distribution. The sizes of the pots simply refers to the “amount” of the SOC pools across land use types.

Rainfall has been reported as the main governing factor of SOM and TN content distribution in Sub-Saharan tropical soils of East Africa^74^. This was affirmed by our result of overall mean high SOC and TN stock especially in Desa’a with high rainfall and low temperature compared to other locations (See Figs. 6 and 7). Observably, SOC stock increased with increasing mean annual precipitation (MAP) and decreased appreciably with increasing mean annual temperature (MAT) (See Figs. 6 and 7). Various authors have reported positive correlation of SOC stock with MAP but with negative correlation with MAT^59,75^. Assefa et al.^59^ reported high SOC stock values in areas with high MAP compared to areas with low MAP in northwest Ethiopia. Estimated SOM content ranging between 0.5–3.0 and 10–13% for tropical soils of Sub-Saharan Africa (high temperature) and temperate soils of Europe/America (low temperature) respectively has been reported^2^. Global distribution of SOC stock follows a pattern, increasing from temperate (cooler) regions to tropical and sub-tropical (hotter) regions^76^. Temperature and precipitation remain the two major environmental factors affecting SOC concentration and stock within the complexity of land use change (LUC)-SOC distribution nexus^77,78^. In our study, MAP has more impact in terms of modulating SOC stock compared to MAT (See Fig. 6). This partly supports our second hypothesis that climate and land use history will serve as possible indicators to modulate the amount of change in SOC stocks.

Another remarkable outlook in this study in terms of drivers of SOC distribution was provided by the clay fraction data on basis of occurrence or proportion. Thus, soils with high clay content recorded high SOC concentration and stock compared to soils with low clay content. This finding is in agreement with the work of^67^ in a study to assess the C:N ratios following land use change in Brazil.

In Cambisols of Hugumburda, Haikihelet and Geregera, the overall mean total sand fraction was high in natural (forest) and semi-natural (exclosure) ecosystems, with high proportions in sub-soil layers in most land use types excluding cropland at Geregera. This is in contrast with the findings of^79^ who reported high sand fraction in grazing land, followed by agroforestry and cropland in Nitosols of southern Ethiopia. In Vertisols of Desa’a, high clay fraction was observed in all the land use types with appreciable increase with depth. Differences in soil types and micro-climatic conditions might be responsible for these variations. Bockheim^80^, Ukaegbu et al.^81^ and Okolo et al.^82^ reported that soils formed on the same parent material within an ecological region are complexly linked to landscape and thus display substantial variations in soil properties. Furthermore, differences in BD was observed across locations in different land use types. Low BD in natural (forests) and semi-natural (exclosures) ecosystems, could be attributed to constant input of high soil organic residues on the upper layer of the soil^83–86^. The contribution of tree roots to the subsoil organic matter (OM) accumulation, including root litter decomposition leads to the decrease in BD in forests^87–89^.

## Conclusions

The total SOC and TN concentrations and stocks were high in natural forest, intermediate in exclosure and grazing land, and low in croplands, and generally decreased with increasing depth in allland use types. Across soil depths and land use types, SOC and TN sequestration was higher in Cambisols than Vertisols, with clay content and MAP rather than C:N ratio alone being the most meaningful indices for SOC storage and soil quality assessment. Conversion of forest to cropland resulted to significant losses of SOC and TN with considerable amount of CO_2_ emission which contributes to change in climate. Exclosure establishment supported restoration of degraded grazing lands with recovery of SOC and TN stocks especially in the topsoil layer (Fig. 7). Thus, exclosure establishment could be a sustainable way to reverse soil fertility decline due to its C and N sequestration potentials. Additionally, more attention needs to be placed not only on the amount of SOC sequestration potential under different ecosystems and land use types in semi-arid area of northern Ethiopia, but also to ensure that they remain undisturbed for long periods of time, with mechanisms to detect differences before commencement of carbon trading schemes.

## Data Availability

The datasets used for this study are available from the corresponding author on reasonable request.
